# Ninth Annual Report of the Managers of the State Lunatic Asylum of the State of New York

**Published:** 1852-05

**Authors:** 


*Ninth Annual Report of the Managers of the State Lunatic Asylum of the State of New York.*

THIS document shows the continued prosperity and usefulness of this, the largest institution for the insane in our country.

The number of patients in the Asylum at the commencement of the year was 429, and there were admitted during the year 366, making a total of 795.

There were discharged during the year, 360: of which number 112 were cured; 15 much improved; 51 improved; 134 unimproved; and 48 died;—leaving in the institution at the close of the year, 435 patients.

The most important suggestion of the managers and superintendent that we note, is a recommendation that the plan of heating and ventilating the establishment be entirely changed.

In the number of this Journal for January last, we noticed the abandonment of the system of forced ventilation, which had been provided for in the erection of the Indiana Hospital for the Insane, and expressed our regret for the move, regarding it as a positive retrogradation. Our conclusions are strengthened by the document before us, in which a system of precisely the same character is proposed to be introduced at great expense in this establishment, which had been erected before the system was introduced into this country.

In reference to it the managers say :—

"With the improved mode of warming by steam, can and should be connected a better and more effectual plan of ventilating the asylum buildings, an improved mode of ventilation is much desired. Experience has shown that the method originally adopted of ventilating by small upward ventilating flues in the partition walls, is inadequate to accomplish the object intended. It is ascertained that some artificial or forced ventilation is necessary to render the atmosphere in public buildings of this character pure and perfectly healthful, and that the means of ventilation should be under such control and regulation that they can be adapted to the various changes of the weather; much greater ventilation being required in some states of the atmosphere than in others. Their forced ventilation can be most advantageously secured by connecting the ventilating flues, from the various rooms of the house with the flue of the chimney which is heated by the fire, by which the building is warmed, thus creating a strong downward draft in the ventilating flues, and which draft may be regulated by registers over the opening flues in the rooms."

The able superintendent, Dr. Benedict, has recommended this plan, and urged it upon the managers, and through them upon the Legislature of the State. E.

